# I Like the Food You Made! Overly Positive Feedback Is Most Likely Given to Those That Want to Excel in a Task and Handle Failure Badly

**DOI:** 10.3389/fpsyg.2022.807958

**Published:** 2022-07-19

**Authors:** Katarzyna Cantarero, Katarzyna Byrka, Aleksandra Kosiarczyk, Dariusz Dolinski

**Affiliations:** Faculty of Psychology in Wroclaw, Social Behavior Research Center, SWPS University, Wroclaw, Poland

**Keywords:** honesty, care, prosocial lying, other-oriented dishonesty, white lies, feedback

## Abstract

In this article, we focus on how people resolve the dilemma between honest feedback and a prosocial lie depending on the context. In a pre-registered study (*N* = 455), we asked participants to choose between telling the blatant truth or lying prosocially regarding a dish made poorly by a stranger. The results showed that participants were most eager to pass on overly positive feedback when the stranger cared about cooking and was very sensitive to negative feedback. Perceived harm in truth telling mediated the relationship between desire to excel in a task with high ability to handle failure and choosing a prosocial lie.

## Introduction

“A white lie is excusable. You cannot forgive someone who speaks the truth when nobody needs that truth.”—Karl Kraus.

When do people choose to tell the truth and when do they prefer to lie^1^ to others? Truth is one of the foundations of many moral systems. The rule to tell the truth can be found in the Bible, Koran, or lay proverbs coming from different cultural backgrounds. Consequently, in many life circumstances, opting for the truth is the default option. For example, imagine that someone asks for your opinion regarding a dish they made. You think that the dish is far from appetizing or esthetically appealing. Imagine then that you know that the cook wants to master the art of cooking and knows how to handle any feedback very well. Giving them your honest opinion for some may seem the most natural thing to do. But what if the person cares about cooking, but you know that they have difficulties in dealing with negative feedback? Would one prefer to tell them the truth? At times a prosocial lie might be the ‘lesser of two evils’ and be preferred by people faced with the dilemma of whether to be honest or use a prosocial lie.

In this article, we focus on how people resolve the dilemma between giving honest feedback and lying prosocially. In the current research, we explored the context of giving feedback regarding a failed dish and tested if participants are less eager to tell the truth depending on whether the recipient wants to excel in a task and how they handle failures. Additionally, we tested the perception of the truth and prosocial lies regarding their deceitfulness, to verify if such polite lies are indeed perceived as lies. The article offers new insights regarding conditions under which people decide to employ a lie rather than honest feedback, which enriches understanding of prosocial deception.

### Characteristic of Prosocial Deception

According to the Truth-Default Theory (TDT), individuals tend to tell the truth by default and think that others do so as well (Levine, 2014). TDT stresses that context determines boundary conditions under which some may decide to deviate from the truth. Levine (2014) argues that trigger events push people to not turn to honest communication. One such triggering event may be when honest feedback can be harmful to the target of the information. Such instances should favor telling a prosocial lie.

While egoistic lies are intended to benefit only the liar, prosocial lies (also referred to as other-oriented lies, and those prosocial lies that are of a lower stake as white lies) are aimed at primarily benefiting another person (DePaulo et al., 1996). In general, prosocial lies are perceived in a different manner to egoistic lies. For example, cross-cultural research shows that such lies are found to be more acceptable that egoistic lies (e.g., Cantarero et al., 2018). What is more, children of ages 9–11 (Cheung et al., 2015) and adults (Cantarero and Szarota, 2017) perceive prosocial lies as lies to a lesser extent. For example, when one person lies that they cannot stay at work to do extra hours, such a lie is perceived as a lie to a higher extent when it is told to please personal interests (e.g., one does not feel like working), than when it is told for the benefit of others (e.g., one knows that a co-worker really needs extra money; Cantarero and Szarota, 2017). Additionally, Levine (2021) showed that lying is perceived as ethical when it prevents unnecessary harm. She suggests that the extent to which truth yields a significant change related to learning or growth is important in deciding whether deviating from the truth is ethical. We hypothesize that when making actual decisions about whether to give honest feedback, or tell a prosocial lie, people also turn to the value of the communication.

### When Is Honest Feedback Desirable?

Brown and Dutton (1995, p. 1293) argued that “people do not always need to know the truth about themselves’ regarding their own self-views.” They reasoned that positive self-views (if not exaggerated) might be beneficial for individuals (see also Taylor and Brown, 1988). One reason for this is that, by doing so, individuals do not get discouraged from what they are doing, or simply feel good about themselves. Additionally, if a person is engaging in a behavior without the goal of maximizing their ability in that behavior, then getting accurate feedback might not be what the person wants (or needs). How do people decide whether to communicate honest feedback or tell a prosocial lie? Lupoli et al. (2017) showed that compassion is one of the factors that drives people to overinflate feedback passed to a recipient. In their study, when participants learned that a person, to whom they were about to pass feedback about a badly written essay, has recently gone through a difficult time, they were more likely to overinflate their evaluation of the essay. Drawing on the works by Levine (2021), we hypothesize that when faced with an actual dilemma about whether to tell the truth or a prosocial lie, individuals take into consideration both whether the information can cause any harm and if it is useful. Accordingly, a prosocial lie is preferred when the target has difficulties in dealing with the blatant truth. Honest feedback is chosen when improvement is being sought by the target of the information. We also hypothesize that individuals are more likely to provide a positive but false evaluation (i.e., a prosocial lie) when the target does not want to improve in an activity or area (to spare him from the blatant truth that the target would probably not use). Importantly, we predict that individuals do not necessarily make unequivocal and objectively beneficial choices for another person. For example, giving accurate feedback is in general beneficial (Ilgen et al., 1979; Sapyta et al., 2005), as it improves the performance of individuals (Mauger et al., 2011; Kingsley Westerman et al., 2018). We hypothesize that people will choose what they *think* is preferable for another person at that time.

In the study by Lupoli et al. (2017), the authors did not test interaction effects between compassion and whether honest feedback is needed by the recipient (e.g., if the target wants to write good essays or not). Additionally, their study did not examine if people were consciously aware of the fact that they were deviating from the truth. In this article, we paid attention to examining if telling a prosocial lie is indeed perceived as a lie by others. This is due to the premise that deviating from the truth for the benefit of another person could be perceived in fact as a mistake, a social norm or a form of self-deception (and thus no longer a lie). In our study, we additionally addressed this question and controlled whether participants perceived the communication that they decided to pass on as a lie (i.e., we tested perceived deceitfulness of the feedback).

To sum up, we focus on how individuals resolve the dilemma of whether to give honest feedback, or to lie prosocially. We expect main effects of willingness to excel in a task and the ability to deal with negative feedback by the target of the feedback. Specifically, we hypothesize that individuals exert a higher preference toward lying when the target does not want to excel in a task than when they want to get better at it. We expect that the preference toward lying is more likely when the target does not handle failure well than when they do. We hypothesize an interaction in that individuals prefer telling prosocial lies the most when the target of feedback does not handle failure well and does not want to excel in a task. We expect that the second context with most frequent preference toward prosocial lying is when the target does not handle failure well and wants to excel in a task. Additionally, we hypothesize that the third context with most frequent prosocial lies is when the target knows how to handle failure and does not want to excel in a task. We expect most frequent honest feedback when the target knows how to handle failure and wants to excel in a task.

Similar to Levine (2021), we think that preference toward prosocial lies is grounded in the desire to protect individuals from harm. We hypothesize that perceived harm in telling the truth mediates the relationship between willingness to excel in a task, ability to deal with failure, and preference toward prosocial lies. Namely, we expect the effect of the experimental manipulation on preference toward prosocial lies to be mirrored in perceived harm of telling the truth. We expect a positive relationship between perceived harm in truth telling and preference toward prosocial lies. Additionally, we tested if prosocial lies are indeed perceived as lies (and not equated to truthful statements), as one could argue that given that politeness is social norm compliance, prosocial lies may no longer be perceived as deception.

## Materials and Methods

The study was pre-registered at aspredicted.org (the anonymized pre-registration is available at https://aspredicted.org/1K2_FY1). All measures, manipulations, and exclusions in the study are disclosed.

### Participants

The sample’s size was determined before conducting the study and before any data analysis. Sample size estimate was calculated using G*Power for *χ*^2^, assuming power (1–*β*) = 0.95, probability level *α* = 0.05, and effect size of *ω* = 0.17. We aimed to reach 450 participants. Four hundred and fifty-five participants residing in the United States took part in this online study *via* Prolific. We invited to participate in the study those who had at least a 95% acceptance rate in previous studies. Three hundred and seventeen women and 127 men (eight participants did not state their gender) participated in the study in exchange for 0.75£.^2^ Ages ranged from 19 to 70 (*M*_age_ = 33.77, SD_age_ = 12.73). No data was excluded.

### Procedure and Materials

First, participants read that the study focused on feelings and opinions that people have toward daily activities (e.g., cooking). Then, they responded to 17 buffer questions regarding cooking and food (e.g., “*I eat at least four meals a day*”). After completing the questionnaire, participants read the instruction that they would be asked to evaluate a dish. Participants were randomly assigned to one of four conditions in a between-subjects design (high vs. low desire to excel in task; high vs. low ability to handle failures). Participants first read: *You will now be asked to give your opinion on a dish that was prepared by another person. You will be able to see what the person wanted to prepare and what the person prepared in the end. This person spent a lot of time preparing the dish.*

Next, participants read one of four descriptions of the person:

1. They do not really like cooking and do not want to get better at it. They do not really handle failure well, which makes the person feel really down.
2. They do not really like cooking and do not want to get better at it. They know how to handle failure really well, which makes the person feel a lot stronger.
3. They care very much about cooking and wish to get better at it. They do not really handle failure well, which makes the person feel really down.
4. They care very much about cooking and wish to get better at it. They know how to handle failure really well, which makes the person feel a lot stronger.

Participants were then presented with information that was meant to strengthen the ecological validity of the set up:

*You can address your opinion directly to that person, who will be able to see your answer. We will send your answer to that person and the information might be published on social media.*

Then, participants saw a picture of a professional dish that the described person supposedly planned to cook followed by a picture of an unattractive dish that the person actually prepared (a cooking fail). We used visual examples of dishes that did not turn as planned and manipulated the extent to which cooking was important to the author of the dish. In Study S1 in the Supplementary Materials, we showed that the cooking fails used in this study were indeed perceived as much worse than the original version of a dish.

Next, participants were asked to choose what they would like to say to the person who prepared the dish: “*The dish looks nice*’ or ‘*The dish does not look nice.*” Each participant received only one out of eight randomly assigned sets of a professional dish and a cooking fail.

Relying on a within-subject design, participants were asked to evaluate the extent to which both types of feedback were useful, good, the truth, and harmful to the person on a 7-point scale (1 = *strongly disagree,* to 7 = *strongly agree*). Participants also evaluated if they considered both of the possible feedbacks a lie using a 1 = *strongly disagree,* to 7 = *strongly agree* scale.

Additionally, participants evaluated whether the dish that the person had prepared resembled the one that the person wanted to prepare, whether the person who had prepared the dish cared much about cooking, whether they felt sympathy toward the person that prepared the dish, and whether they had previously seen the picture of the dish. Responses were collected using a 1 = *definitely not*, to 5 = *definitely yes* scale. We also asked how participants thought that the person would react after having heard the feedback coming from them using a 1 = *The person will feel very bad*, to 5 = *The person will feel very good* scale.

At the end of the study, we gathered demographic data and debriefed participants.^3^

### Results

In general, participants preferred telling the truth (54%) to prosocial lying (46%), *χ*^2^ (1, *N* = 455) = 3.01, *p* = 0.083, and *ω* = 0.08, yet this difference did not reach the conventional *p* < 0.05.^4^ The results showed that manipulating whether the described person can handle feedback affected the preference of a lie over truth, *χ*^2^ (1, *N* = 455) = 12.72, *p* < 0.001, and *ω* = 0.17. When the person could handle failure well, individuals preferred to tell them the blatant truth (62%) over prosocial lying (38%). The reverse was true when they were bad at handling failures with participants showing a preference for lying more frequently (55%), than toward telling the truth (45%). There was no main effect of whether the described person wanted to excel in cooking or not, *χ*^2^ (1, *N* = 455) = 0.19, *p* = 0.662, and *ω* = 0.02. When the person wanted to excel, the truth was preferred more (53%) to lying (47%), similar to when the person did not want to excel, with 55% preferring the blatant truth, as compared to 45% of preference toward lying.

**Figure 1:**
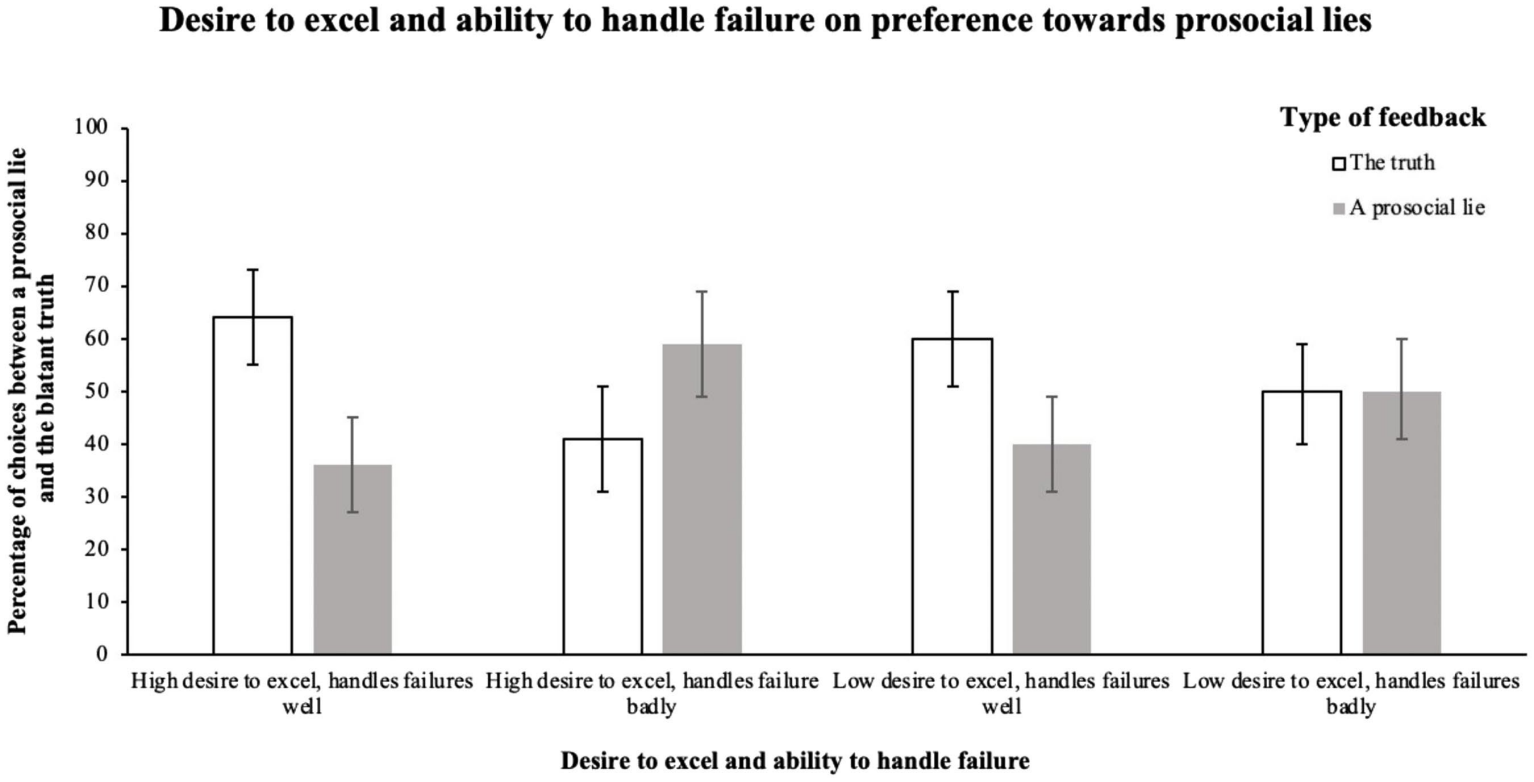
The effect of the desire to excel in a task and ability to handle failure on preference toward overly positive feedback. Figure shows percentage of the preference toward prosocial lies within each condition. Error bars represent 95% confidence intervals.

Most interestingly, there was a significant interaction between the variables, *χ*^2^ (3, *N* = 455) = 14.76, *p* = 0.002, and *ω* = 0.18. When the cook could handle failure well, individuals preferred telling them the blatant truth both when they wanted to excel in cooking (64% vs. 36% preferred prosocial lying) and when they did not want to excel in cooking (60% vs. 40% preferred prosocial lying). The proportion of preference toward prosocial lying between the latter two conditions did not differ from each other. When the cook could not handle failure well and was not interested in excelling in cooking, individuals preferred telling the blatant truth less than in the previous two cases, yet there was no difference in frequency of choices between lying (50%) and telling the truth (50%). The highest preference toward prosocial lying (59%) compared to telling the blatant truth (41%) was observed in the condition where the cook did not know how to handle failure well but wanted to excel in cooking (Figure 1).

**Figure 2:**
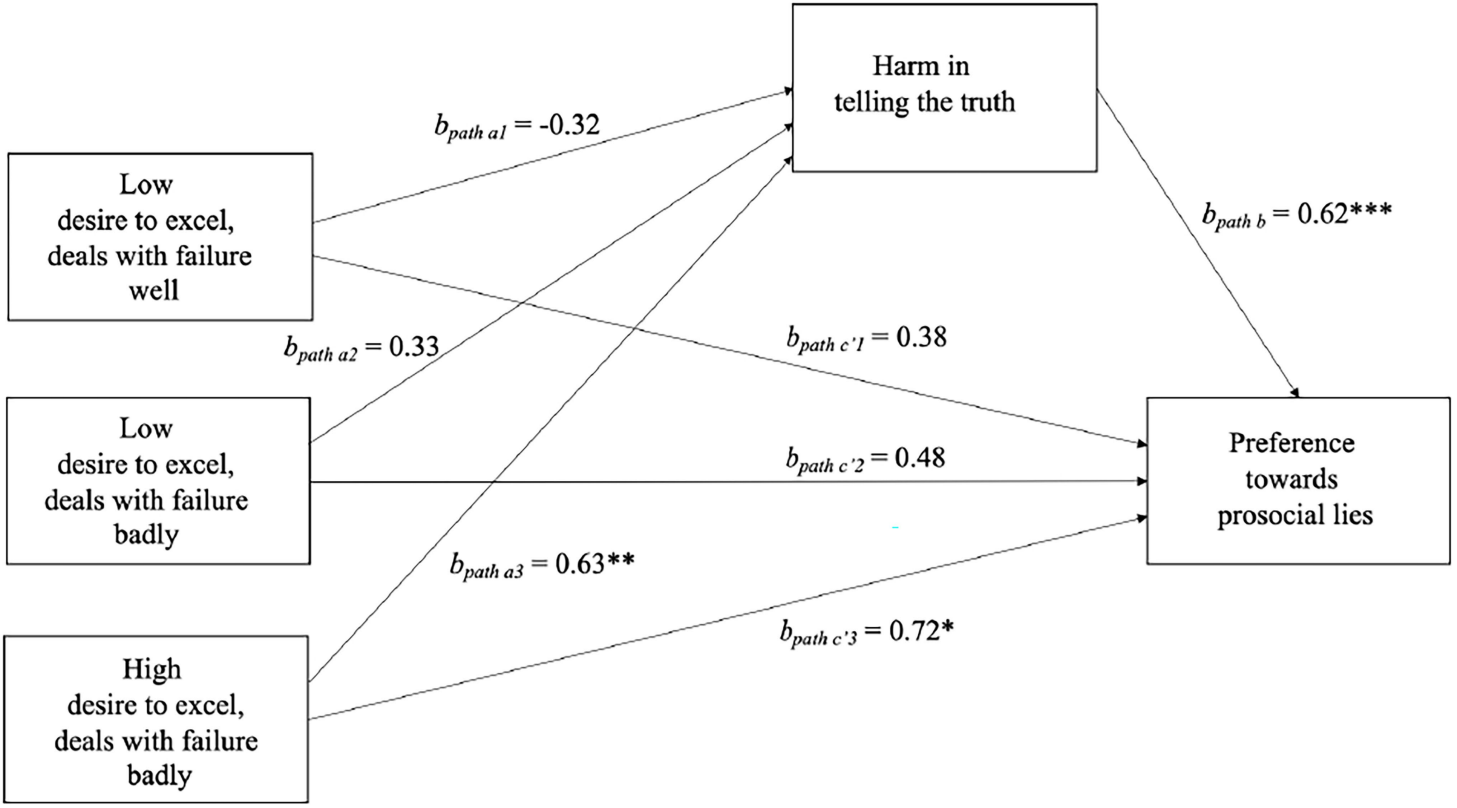
Mediation model testing that harm in telling the truth mediates the effect of desire to excel and ability to deal with feedback on preference toward prosocial lies. In this model, high desire to excel in a task and dealing with feedback well is the reference group, ^*^p < 0.05, ^**^p < 0.01, ^***^p < 0.001.

Next, we conducted a mediation analysis using Model 4 Hayes PROCESS Macro (Hayes, 2013) with a bias-corrected bootstrapping procedure (10,000 samples). The experimental manipulation was introduced as a multicategorical IV, perceived harm in telling the truth was the mediator, and preference toward prosocial lying was the DV in the model. We used indicator coding with high desire to excel and high ability to handle failure as reference group.^5^ The only significant relative direct effect on the preference of lying was in the condition of desire to excel in the task when dealing with failure badly compared to the remaining conditions, *b*_path *c′3*_ = 0.72, *p* = 0.020, 95% CI [0.12, 1.32].^6^ This condition was related to higher preference toward prosocial lying. The same condition, as compared to others, was related to higher perception of harmfulness of truth telling, *b*_path *a3*_ = 0.63, *p* = 0.004, 95% CI [0.20, 1.06]. The other conditions did not relate to perceived harmfulness of truth telling in a significant way. When both the experimental manipulation and perceived harm in truth telling were entered into the equation, perceived harm in truth telling significantly predicted positively choosing a lie over the truth, *b*_path *b*_ *=* 0.62, *p* < 0.001, and 95% CI [0.47, 0.76]. The relative indirect effect was statistically significant only in this condition, *b*_path *a3b*_ = 0.39, bootSE = 0.14, and bootCI [0.13, 0.70] (Figure 2).

We compared perceived deceitfulness (where 1 = *definitely not a lie* and 7 = *definitely a lie*) of the two types of feedback that could be communicated using paired t-tests. This way we could examine if overly positive feedback and the blatant truth differ in being perceived as a lie. Higher scores of overly positive feedback would suggest that it is treated more as a lie, than the blatant truth. The results showed that telling the person that what they prepared looks nice was perceived as more deceitful (*M* = 5.40, SD = 1.68), than telling them that the dish does not look nice (*M* = 2.31, SD = 1.47), *t*(453) = 22.73, *p* < 0.001, and *d* = 1.07.^7^ Similarly, one-sample *t*-test allowed us to test if perception of deceitfulness falls above the mid-point of the scale, with (4) as the tested value, which would additionally suggest that overly positive feedback is perceived as a lie. The results showed that the evaluation of deceitfulness of the overly positive feedback was significantly above the mid-point of the scale (*M* = 5.40, SD = 1.68), *t*(452) = 17.71, *p* < 0.001, and *d* = 0.83, suggesting that it was perceived as a lie.

## Discussion

The findings of this research are in line with the TDT that argues that individuals prefer to tell the truth unless there are triggering events that push individuals’ preference toward deception (Levine, 2014). We show that manipulating whether the target of information needs honest feedback and how they can handle it influences the decision of whether to be honest or to lie. When the target did not know how to handle failure, the likelihood of telling them the blatant truth diminished. This result is in line with Lupoli et al. (2017) who showed that compassion influences choosing prosocial lying. We found that among those that want to excel in a task, it is especially those that handle failure well that were more likely to be given honest feedback. We think that it may be due to a general preference to be honest (e.g., Abeler et al., 2019). It seems that the situation with low desire to excel in a task did not present itself as a sufficient trigger to push individuals to deviate from the truth. It is possible that compassion plays a vital role as a trigger of deviating from the truth. Together with the mediating role of harm attributed to telling the truth, the results corroborate the findings pointing to the determining role of consideration of harm for moral judgments and behavior (e.g., Schein and Gray, 2018).

We also did not find that it is above all those who do not want to excel in a task and do not handle failure well that most frequently receive overly positive feedback. The relationship between the two factors proved to be more complex than we expected. Lying comes with the price of having to juggle positive self-views as an honest person and possible benefits related to lying (Mazar et al., 2008). Our study showed that only when the target wants to excel in a task and does not know how to handle failure, are people more willing to lie prosocially toward them. This suggests that individuals most likely want to deviate for the benefit of another person when it is really worth it. Although we did not ask participants to provide a more specific reason for their decision, it may be that by passing on overly positive feedback, individuals did not want to discourage the motivated individual from further work toward reaching excellence in the task. This possible explanation could be tested in future studies. Additional analyses showed that the prosocial lie was perceived as more deceptive than telling the blatant truth. This suggests that false and overly positive feedback should not be reduced to social norm compliance and simple acts of politeness.

### Limitations and Directions of Future Studies

There are some limitations of the conducted study that should be mentioned. First, we preferred to measure the DV right after the experimental manipulation, to make sure that the result can be attributed to the manipulation and not be inflated by having marked additional measures in between. The variable we chose for the mediation analysis was the one that was most theoretically sound, but it was not measured in chronological order (i.e., first the IV, next the mediator, and finally the DV) and such alignment would be optimal to test causal relationships.

We recognize that limiting sample to Northern American participants enrolled at Prolific is a drawback of the present study. Research shows that there are cultural differences in both attitudes toward lying (e.g., Cantarero et al., 2018) and dishonest behavior (e.g., Cohn et al., 2019). Preference for a prosocial lie vs. telling the truth could vary across cultures and testing such possible differences seems an exciting line of future research. Initial findings by Giles et al. (2019) suggest that members of individualistic cultures may favor more strongly telling blatant truth to prosocial lying. Additionally, we tested preference for overly positive feedback vs. the blatant truth in a virtual context. Cantarero et al. (2017) found that when individuals were asked to give their opinion regarding a picture, they provided more positive feedback to the alleged author of the work in person, than when the opinion was gathered privately in writing. It would be interesting to see if in face-to-face communication, desire to excel and ability to handle failure exerted the same effect on preference toward prosocial lies as in the online setting. Here, we limited our design to giving feedback anonymously to a stranger. Future studies could include the type of relation and whether the information is conveyed anonymously or not, as these factors relate to lying prosocially (e.g., DePaulo and Kashy, 1998; Levine, 2021).

The two options of the dilemma (blatant truth vs. a prosocial lie) do not exhaust the range of possible reactions to situations where one is asked for feedback (e.g., one could try to omit responding, resort to irony, or provide a blurry response that does not answer the question). In this study, however, we wanted to focus only on the dilemma between blatant honesty and a prosocial lie. Future studies may want to explore preferences toward the two options together with the gray zone in between and test how individuals perceive forms of feedback other than telling the blatant truth to a target (e.g., concealment, irony, and half-truths).

Cantarero and Szarota (2017) point out that other-oriented lies are seen as lies to a smaller extent, and Levine and Schweitzer (2014) found that prosocial lying is seen as more ethical than self-centered truth telling. It is worthwhile to expand on the determinants of when a lie is perceived as less of a lie. In our research, we measured whether individuals perceived communication that intentionally misled another person for their benefit as lying and found that these acts were not perceived as equal to truth telling. This indicates that although a prosocial lie is less of a lie, it still remains a lie.

To sum up, our study employed a social decision-making dilemma to show that when deciding to choose between honest feedback and prosocial lying, people most likely opt for prosocial lies when the target wants to excel in a task but has trouble dealing with failure. What is more, the preference for prosocial lying is most likely due to the perception of harm attributed to truth telling. This is the first study that relies on actual behavior and tests the role of target’s desire to excel and ability to handle failure on preference toward telling them prosocial lies. Results of this study could be applied to academic or work-related environment in general. If a student, or a worker, who wants to excel in a task, would like to make sure that they receive honest feedback on their work, they should try to convey that they have no trouble in handling failures. Otherwise, their interlocutor may want to spare them from unnecessary harm and overinflate positive feedback.

## Author’s Note

Parts of this article can be found in a pre-print version at the https://psyarxiv.com/jm7y4.

## Data Availability Statement

The datasets presented in this study can be found in online repositories. The names of the repository/repositories and accession number(s) can be found at: https://osf.io/6rjka/?view_only=8319d26515354a959d552ab20c7efdb2.

## Ethics Statement

The studies involving human participants were reviewed and approved by Faculty of Psychology in Wroclaw Committee of Ethics of Scientific Research at SWPS University, number 02/P/11/2018. The patients/participants provided their written informed consent to participate in this study.

## Author Contributions

KC, KB, AK, and DD designed the main study and the supplementary study, and contributed to the writing of the manuscript. KC conducted the studies and the analysis. All authors contributed to the article and approved the submitted version.

## Funding

This work was supported by a research grant awarded by the National Science Center in Poland to KC (2020/39/B/HS6/02196) and by research grants awarded by the Ministry of Science and Education in Poland to KB (BST/Wroc/2018/A/2) and to DD (BST/Wroc/2018/A/3).

## Conflict of Interest

The authors declare that the research was conducted in the absence of any commercial or financial relationships that could be construed as a potential conflict of interest.

## Publisher’s Note

All claims expressed in this article are solely those of the authors and do not necessarily represent those of their affiliated organizations, or those of the publisher, the editors and the reviewers. Any product that may be evaluated in this article, or claim that may be made by its manufacturer, is not guaranteed or endorsed by the publisher.

## Supplementary Material

The Supplementary Material for this article can be found online at: https://www.frontiersin.org/articles/10.3389/fpsyg.2022.807958/full#supplementary-material

## Footnotes

^1^In this article we use the terms: lying, deception and dishonesty interchangeably and define prosocial lies as lies that are primarily aimed at benefitting another person.

^2^The study lasted on average less than 6 min, mode was 3 min.

^3^We also asked an attention check question. One person did not answer it correctly. Fourteen participants did not respond to this question. As pre-registered, we checked if the results differed when excluding those that failed to reply to the attention check question. The results remained consistent regardless of whether these 15 participants were excluded. The main effect of dealing with feedback was significant, *χ*^2^ (1, *N* = 440) = 12.92, *p* < 0.001, and ω = 0.17 as well as the interaction with the desire to excel in cooking, *χ*^2^ (3, *N* = 440) = 15.32, *p* < 0.001, and ω = 0.19.

^4^This general analysis was not pre-registered. In the Supplementary Materials, we present the results of the manipulation check. Participants correctly recognized whether the described person wanted to excel in a task or not, but we were not successful in creating a situation that differed in perception of usefulness of the information.

^5^We used this condition as the reference group as we hypothesized and found that this group could be treated as baseline as it was related to the highest rate of honest feedback.

^6^Detailed results are presented in Supplementary Table S2 in Supplementary Materials.

^7^Similarly, telling the person that what they prepared looks nice was perceived less as the truth (*M* = 2.79, SD = 1.83), than telling them that the dish does not look nice (*M* = 5.49, SD = 1.58), *t*(454) = 22.73, *p* < 0.001, and *d* = 1.07.
