# Supplementary material for: I Like the Food You Made! Overly Positive Feedback Is Most Likely Given to Those That Want to Excel in a Task and Handle Failure Badly
Source: Front Psychol. 2022 Jul 19;13:807958. doi: 10.3389/fpsyg.2022.807958 (PMC9344058; doi:10.3389/fpsyg.2022.807958)
Supplement: Supplementary file 1 [file Data_Sheet_1.pdf]

Supplementary Materials

I Like the Food You Made! Overly Positive Feedback Is Most Likely Given to Those That Want to Excel in a Task and Handle Failure Badly

Study S1

In Study S1 we tested whether the material used in the Main Study that presented an alleged dish prepared by a person (a cooking fail) was indeed perceived worse to its 'as should be' versions (i.e., what the person tried to prepare).

Method

Participants. One hundred sixty-two mTurk employees, 86 women and 74 men (two participants did not state their gender) participated in a study in exchange for 0.30 \$. Ages ranged from 20 to 68 (M_{age} = 36.52, SD_{age} = 12.30). Sensitivity analysis with $(1 – \beta) = .80$, alpha 0.5, two-tailed, indicated that the overall sample size allows to detect an effect size of $d = .45$ with critical t of 1.97.

Procedure and Materials. First, participants read that the study focused on attitudes towards food and cooking. Then, they provided socio-demographic data. Participants were asked to evaluate a dish presented in a photo (we used the pictures presented to participants in Study 2 and Study 3). Each participant saw only one of sixteen pictures (there were in total 8 cooking fails and 8 well prepared, professional dishes).

Participants were then asked to answer five questions regarding the dish. The questions were presented in random order. We asked to rate the extent to which the dish looks nice, is tasty, whether it required a lot of effort, whether it required high cooking skills and whether it was a result of following the recipe from A to Z. Responses were collected using a 7-point scale (1 = *strongly disagree,* to 7 = *strongly agree*). Participants were debriefed at the end of the study.

Results and Discussion

We calculated mean replies regarding the five types of evaluations separately for cooking fails and professional dishes. We next compared mean evaluations of the cooking fails to the professional dishes using independent t-tests. Cooking fails were evaluated as

looking significantly worse than the professional dishes, $t(158.99) = 10.62$, $p < .001$, $d = 1.68$. Professional dishes looked more tasty, $t(158.73) = 7.82$, $p < .001$, $d = 1.23$ and they were perceived as requiring more effort $t(159) = 5.22$, $p < .001$, $d = 0.83$ and higher skills $t(159) = 4.42$, $p < .001$, $d = 0.69$ than the cooking fails. Finally, participants perceived the professional dishes being much more a result of a followed recipe than the cooking fail, $t(159) = 4.56$, $p < .001$, $d = 0.72$. The means and standard deviations of the evaluations of the cooking fails and professional dishes are presented in Table S1.

Table S1

Evaluations of cooking fails and professional dishes Study S1.

Type of evaluation	Cooking fail		Professional dish	
	M	*SD*	*M*	*SD*
Looks nice	2.76	1.78	5.47	1.45
Is tasty	3.08	1.75	5.07	1.48
Required effort	3.96	1.68	5.28	1.48
Required high skills	2.70	1.64	3.90	1.82
Follows the recipe	3.53	1.77	4.81	1.77

Note. $N = 160$.

The results of this study showed that the cooking fails we chose for the study were perceived as much worse in terms of physical appearance and ascribed quality of taste. In a between-subject design we showed that the cooking fails also received lower scores than the professional dishes regarding perception of required effort and skill. These results elevate our trust in that when participants chose to communicate that the dish looked nice, s/he was sending such a feedback regarding a dish that in fact was evaluated as far less attractive than the professional dish.

Manipulation Check Main Study

First, we checked whether participants adequately perceived whether the person who prepared the dish differed in the extent to which they cared about cooking depending on the experimental condition. In the condition with high desire to excel in cooking, the person was

perceived more (*M* = 3.98, *SD* = 0.83) as a cooking affiliate than in the low desire to excel in cooking condition (*M* = 1.97, *SD* = 0.82), $t(451) = 25.84$, $p < .001$, $d = 0.83$.

In general, participants thought that the dish that was prepared did not resemble the one that the person wanted to prepare (*M* = 2.49, *SD* = 1.09) and they declared not having seen the pictures previously (*M* = 1.66, *SD* = 0.86) [1].

Results of a mixed factorial ANOVA with perceived usefulness of information as DV, type of information (blatant truth vs. prosocial lie) as within-subjects factor and the experimental manipulation (desire to excel in task high vs. low x ability to handle failures high vs. low) as between-subject factors showed main effect of the type of information, $F(1, 450) = 277.73$, $p < .001$, partial $\eta^2 = .38$. Lying (*M* = 3.24, *SD* = 1.70) was perceived as less useful than truth telling (*M* = 5.49, *SD* = 1.58). There was no between-subjects effect of either the desire to excel in cooking ($p = .972$), nor its interaction with knowing how to handle failures ($p = .910$). The results showed also no interaction with the type of feedback and knowing how to handle failure ($p = .557$), the desire to excel in cooking ($p = .645$) or the interaction of these variables ($p = .797$).

These results suggest that although participants correctly recognized whether the described person wanted to excel in a task or not, we were not successful in creating a situation that differed in perception of usefulness of the information.

Detailed results regarding the mediation analysis are presented in Table S2

---

[1] There were no significant differences between the conditions and their interaction regarding resemblance of the dish to what the person wanted to prepare ($p_s \geq .140$), having seen the pictures previously ($p_s \geq .187$).

Table S2.
Results of mediation analysis.

IV	Relative direct effect of IV on the DV (c’)	Effect of IV on the mediator (a)	Relative indirect effect (ab)
High desire to excel, handles failure badly	0.72*(.31), [0.12, 1.32]	0.63**(.22), [0.20, 1.06]	0.39(boot se .14) boot [0.13, 0.69]
Low desire to excel, handles failure well	0.38(.30), [-0.20, 0.96]	-0.32(.21), [-0.73, 0.10]	-0.19(boot se .13) boot [-0.47, 0.06],
Low desire to excel, handles failure well	0.48(.30), [-0.11, 1.06]	0.33(.21), [-0.09, 0.75]	0.20(boot se .14) boot [-0.05, 0.49],

Note. *<.05, **<.01, ***<.001. Paths a, c’ and ab are presented with standard errors in parenthesis. Square brackets present 95% confidence intervals. Effect of mediator on DV (b) was significant 0.62***(.07), [0.47, 0.76].

Additional Table Main Study

Table S3
Frequencies of preference towards overly positive feedback depending on whether the target handles failure well and wants to excel in cooking.

Type of evaluation	Blatant truth		Overly positive feedback	
	n	%	*n*	%
High desire to excel, deals with failure well	72	64	40	36
High desire to excel, deals with failure badly	43	41	62	59
Low desire to excel, deals with failure well	74	60	49	40
Low desire to excel, deals with failure badly	57	50	58	50

Note. $N = 455$.

Materials Used in the Main Study

We used pictures of a chocolate turkey, Star Wars pancakes, Sour Patch cake and chocolate ghosts that can be reached for example here:

https://www.awesomeinventions.com/expectation-vs-reality-cooking-fails/?__cf_chl_jschl_tk__=pmd_WE5qKbKvhYKwxcgPpWrHstpPW94TqptdKjzI7CHJZGQ-1629817929-0-gqNtZGzNAfujcnBszQc9

We used pictures of a pancake bunny, that can be found for example here:

https://brainberries.co/funny/cooking-fails-expectations-vs-reality/?utm_campaign=bb_revc_inter_desk&utm_content=45244&utm_medium=cpc&utm_source=bb_revc_inter_desk&utm_term=5523

We used pictures of hummus pumpkins with salted sticks that can be found for example here:

https://www.sadanduseless.com/funny-kitchen-flops/

We used pictures of a pumpkin cake that can be found for example here:

https://definition.org/cooking-fails/1/
